# Assessment of walkability and walkable routes of a 15-min city for heat adaptation: Development of a dynamic attenuation model of heat stress

**DOI:** 10.3389/fpubh.2022.1011391

**Published:** 2022-11-04

**Authors:** Yu Wang, Bao-Jie He, Chong Kang, Li Yan, Xueke Chen, Mingqiang Yin, Xiao Liu, Tiejun Zhou

**Affiliations:** ^1^School of Architecture and Urban Planning, Chongqing University, Chongqing, China; ^2^Institute for Smart City of Chongqing University in Liyang, Chongqing University, Liyang, China; ^3^Key Laboratory of New Technology for Construction of Cities in Mountain Area, Ministry of Education, Chongqing University, Chongqing, China; ^4^State Key Laboratory of Subtropical Building Science, South China University of Technology, Guangzhou, China; ^5^School of Civil Engineering and Architecture, Southwest University of Science and Technology, Mianyang, China; ^6^School of Architecture, South China University of Technology, Guangzhou, China; ^7^Architectural Design and Research Institute Co., Ltd., South China University of Technology, Guangzhou, China

**Keywords:** extreme heat, 15-min city, thermal comfort, dynamic attenuation model, UTCI, ENVI-met

## Abstract

Actively addressing urban heat challenges is an urgent task for numerous cities. Existing studies have primarily developed heat mitigation strategies and analyzed their cooling performance, while the adaptation strategies are far from comprehensive to protect citizens from heat-related illnesses and deaths. To address this research gap, this paper aims to enhance people's adaptation capacity by investigating walkability within fifteen-minute cities (FMC). Taking cognizance of thermal comfort, health, and safety, this paper developed a dynamic attenuation model (DAM) of heat stress, along with heat stress aggravation, continuance, and alleviation. An indicator of remaining tolerant heat discomfort (*R*_*t*_) was proposed with the integration of the Universal Thermal Climate Index (UTCI) to assess heat-related walkability. Following an empirical study among 128 residents in Mianyang, China, and assessing four levels of heat stress, the maximum tolerant heat discomfort was determined to be 60 min. Furthermore, the DAM was applied to an FMC with 12 neighborhoods in Fucheng, Mianyang, China. The results indicate that for each neighborhood, the street was generally walkable with an *R*_*t*_ ranging between 15 and 30 min, after walking for 900 m. A population-based FMC walkability was further determined, finding that the core area of the FMC was favorable for walking with an *R*_*t*_of 45–46 min, and the perpetual areas were also walkable with an *R*_*t*_of 15–30 min. Based on these results, suggestions on the frequency of public services (frequently used, often used, and occasionally used) planning were presented. Overall, this paper provides a theoretical model for analyzing walkability and outlines meaningful implications for planning heat adaptation in resilient, safe, comfortable, and livable FMCs.

## Introduction

Cities are now the main human settlement and urbanization will remain mainstream in the coming decades, especially in Asian and African nations (1). Whilst urban living infrastructure is getting better and living quality is improving, the upward trend of urbanization is accompanied by various challenges such as climate change, environmental deterioration, traffic congestion, housing shortage, urban noise, rapid economic development, and inadequate health facilities (2–4). To address such challenges, the UN Sustainable Development Goals (SDGs), particularly Goal 11, suggested the creation of sustainable cities and communities to ensure cities are inclusive, safe, resilient, and sustainable. Goal 11 is interconnected with other goals such as Goal 13 on climate action, where changing climate is a macro driver of various disasters such as urban flooding, extreme heat, and air pollution. Problems such as urban flooding and air pollution have already been widely recognized, and various interventions and actions in aspects of policy, finance, programs, and initiatives have been implemented to address them. However, urban heat challenges have not received enough attention from society and limited measures have been adopted in practice (5).

Urban heat is the combined effect of heat waves and urban heat islands (UHIs) associated with climate change and urbanization (6). Numerous climate-related research among urban physicists and meteorologists have revealed the formation, aggravation, and drivers of urban heat (7–9). Their significant impact on environmental, economic, social, and health aspects has also been widely reported (10). However, urban heat has not been widely recognized as an emergent disaster, although it has caused excessive deaths (11–13). The extreme heat event in August 2003 killed more than 70,000 Europeans (14). In 2015, about 3,000 French lost their lives because of extreme heat events (15). In 2019 and 2020, the extreme heat killed 2,000 people each year in England (16). Urban heat has now emerged as a silent killer, causing higher mortality than any other climate-related disaster (17). In the coming decades, heat waves are projected to be more frequent, intense, and longer. While at the same time, contiguous modifications of natural environments during urbanization will further aggravate UHIs in dense and prominent areas associated with heat-absorption and storage material use, nature-based infrastructure reduction, anthropogenic heat release, and increased urban density (18). Synergies between heat waves and UHIs can possibly further aggravate heat-induced impacts (19). In general, addressing the urban heat challenge is an urgent task, not only for the current situation but also in the context of constant climate change.

**Figure 1:**
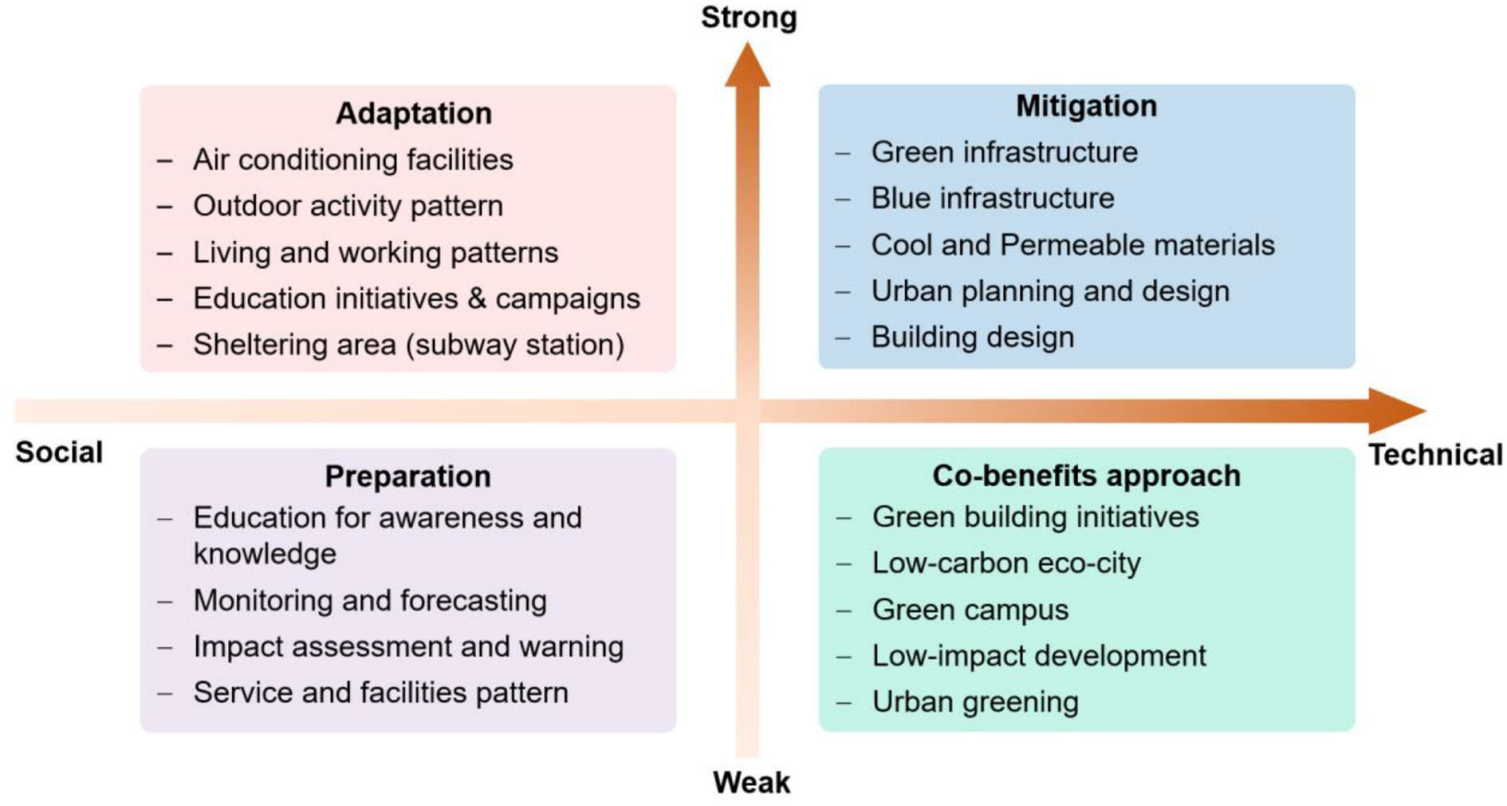
Social-technical solutions to urban heat challenges (20).

To address heat-related challenges, He et al. (20) developed a comprehensive heat-resilient framework consisting of heat preparation, heat mitigation, heat adaptation, and a co-benefits approach (20). Heat preparation refers to the development of heat monitoring, impact assessment, heat health information transfer, and education system to improve heat impact prediction capability and to increase people's awareness and knowledge of what heat impacts are and how to address them. Heat mitigation is focused on heating source dissipation and cooling source enhancement through green and blue infrastructure (21, 22), innovative materials (reflective, retro-reflective, and permeable) (23, 24), urban ventilation (25, 26), and shading structures (27, 28) (Figure 1). Although heat adaptation does not alleviate heat severity from the source, it is currently the most effective approach to protecting people from heat-related illnesses and deaths through the adoption of air-conditioning facilities, behavioral and operational change, and activity rescheduling. The co-benefits approach has been thought of as a win-win strategy in tackling climate change issues by employing the additional benefits of other projects and initiatives for heat preparation, mitigation, and adaptation (29).

Heat adaptation is complex depending on individuals' body heat tolerance, personal behaviors, enterprise operation, and governmental department adaptation. First, the body can resist heat stresses to an extent, which is also known as body thermoregulation. The heat resistance capacity varies with demographic characteristics (such as gender, health, and age), where children and older adults are weak in resisting heat stresses (30). Second, people can change their daily functions such as outdoor activities, work/study, transportation, sleep/rest, and diet in types, time, and duration to adapt to heat (31). In particular, laborers and farmers who need to work outdoors can take their breaks in air-conditioned places or cooling centers during extremely hot temperatures (32). People can select heat-resilient walking routes if outdoor activities are necessary (33). Third, enterprises could upgrade service or operation patterns to ensure emergency systems and public facilities work well under extreme conditions (34). For instance, electricity suppliers could improve production and upgrade the grid to meet increasing energy demands associated with the increase in air-conditioning systems for cooling and overcome grid failure due to extreme heat (34). Fourth, governmental departments could improve their responses for dealing with urban heat by recognizing challenges, formulating policies, setting up financial schemes, and supporting pilot projects and initiatives, to overcome the lack of policies and formal governance (35). However, knowledge of how to improve heat resilience capacity in these four aspects is still a big research gap.

Many global cities are exploring 15-minute city (FMC) models aiming to promote the creation of sustainable and livable communities, neighborhoods, and 15-minute community-life circles with three primary goals of inclusion, health, and safety (36). The FMC initiative is an active response to SDG 11 on sustainable cities and communities to make cities and communities inclusive, safe, resilient, and sustainable. Various FMC frameworks have been piloted in cities across the world including Australia, China, Colombia, France, Italy, and the United States (36). Whilst such frameworks consider factors such as residence, job opportunities, transport, services, and entertainment, the improvement of heat resilience capacity has not been considered, or at least the interlinkage between FMC and extreme heat, a macro background of numerous cities, has not been well considered. It is essential to integrate heat resilience into FMC planning. Until now, the only work on the integration of heat resilience and FMC has been conducted by Chen and He (37) by developing a framework to integrate heat adaptation into FMC through the identification of heat impacts, recognition of heat-induced impact, documentation of influential factors, the suggestion of heat adaptation strategies, and optimization of adaptation (37).

To overcome the research gap relevant to heat resilience and its integration into FMC, this paper aims to investigate the heat-related walkability within the FMC. The consideration of heat impacts on walkability adds new knowledge to people's understanding of neighborhood walkability based on facilities, public services, and demographic characteristics (38, 39). In particular, this paper developed a dynamic attenuation model (DAM) of heat stress to assess heat tolerance when people are walking along specific routes. The model was further applied to an FMC in Fucheng District, Mianyang, China, to identify the heat stresses and the most walkable paths. Overall, this paper can better support stakeholders to integrate heat resilience into FMC and improves the understanding of walkability amid heat stresses.

## Heat-related adaptation and heat tolerance

Based on the three primary goals of inclusion, health, and safety in the FMC, the targets of heat resilience improvement are expected to be inclusion, health, and safety. Namely, the fundamental target of integrating heat resilience into FMC is to improve all possible individuals' capacity of avoiding heat-related impacts on thermal comfort, heat stresses, illnesses, and deaths through heat preparation, mitigation, and adaptation. Beyond this, the integration can further enhance economic growth and social equality by improving thermal environments. There have been numerous indicators to assess the thermal conditions of outdoor environments, such as air temperature, surface temperature, heat stress, and thermal comfort. Among them, thermal comfort, which refers to individuals' subjective feelings of thermal environments, has been widely accepted to assess if the environment is suitable for activities such as working, walking, and standing.

### Thermal comfort and assessment indicators

Subjective feelings are the combination of physical, physiological, and psychological responses to thermal environments (40). The psychological response is associated with people's experience and expectations during the perception and reaction toward heat senses. The physiological response is assessed by the thermo-adaptive approach and governed by the human heat balance, under the change of physical heat exposure. The physiological responses can be measured by the body core temperature, sweat rate, and skin temperature (41). The physical response is relevant to the built form and microclimatic environments which can affect the individuals' physiological responses. Many recent studies have linked physical environments in terms of sound and visual landscape with psychological responses (41). In addition, thermal comfort can be regulated by social and behavioral characteristics (42, 43).

Studies on thermal comfort have been conducted for over 100 years, and the PMV-PPD was the first thermal comfort evaluation indicator developed by Fanger to define comfort, by considering heat-balance equations and empirical studies about skin temperature. It presents a simple method of a seven-point scale from cold (−3) to hot (+3) to survey respondents' feelings (44). Following Fanger, more than 160 thermal comfort assessment indicators have been proposed (45). The WetBulb Globe Temperature (WBGT) is a comprehensive temperature index to evaluate the impacts of temperature, humidity, and solar radiation on people (45). In general, the larger the WBGT value, the harder it is for the human sweat to evaporate, and the lower the body's ability to dissipate heat. This indicator has been used to inform industrial hygienists, athletes, sporting events, and the military of suitable situations for exercise. For instance, during hot climates in the US, when the WBGT is below 22°C, individuals can conduct normal activities, and when the WBGT increases to 28.8°C, there should be limited intense exercises and total daily exposure to heat and humidity. When the WBGT increases to 31.0°C, all exercises should be canceled (46).

The physiological equivalent temperature (PET) and the Universal Thermal Climate Index (UTCI) are found to be the most accurate indicators of outdoor thermal comfort assessment. Both PET and UTCI are a function of environmental parameters (e.g., air temperature, relative humidity, wind speed, solar radiation) and personal behaviors (e.g., clothing, metabolic rate). Both have been classified into different grades of heat stress according to different values (Table 1). For instance, a PET value ranging between 35 and 41°C indicates strong heat stress for western/middle European countries (47). However, there are some differences in people's feelings toward heat stresses after long-term adaptation, where a PET ranging between 38 and 42°C indicates strong heat stress for people from subtropical areas (48). In comparison, the UTCI has different levels of heat stress, where a UTCI value ranging from 38 to 46°C indicates a very strong heat stress (49). Both PET and UTCI have a significant correlation with extreme heat-induced mortality. For example, Matzarakis et al. (47) analyzed the relationship between heat stress and mortality from 1970 to 2007, finding that in Vienna, at 14:00 central Europe time, the mortality at a PET value of 41°C increased to 8.9% on the first day and reached the maximum value of 27.4% on the fourth day, about 3.42 and 10.53 times, respectively of the average mortality level (2.6%) (47). Furthermore, by analyzing the relationship between daily mortality and heat stress in eight Polish cities between 1975 and 2014, Kuchcik found that the risk of death increased to 10–20% in most cities when the UTCI exceeded 32°C, and to 25–30% when the UTCI crossed 38°C (50).

**Table 1:** Heat stress and thermal comfort indicators of PET and UTCI (47–49).

**Stress category**	**PET range for Western/Middle European countries (°C)**	**PET range for subtropical regions (°C)**	**UTCI range (°C)**
Extreme cold stress	<-4	<14	<-40
Very strong cold stress	–	–	−40 to −27
Strong cold stress	4–8	14–18	−27 to−13
Moderate cold stress	8–13	14–22	−13 to−0
Slight cold stress	13–18	22–26	0–9
No thermal stress	18–23	26–30	9–26
Slight heat stress	23–29	30–34	–
Moderate heat stress	29–35	34–38	26–32
Strong heat stress	35–41	38–42	32–38
Very strong heat stress	–	–	38–46
Extreme heat stress	>41	>42	>46

### Spatiotemporal heterogeneity of thermal environments

Heat intensity of outdoor environments is determined by heat sinks and sources which are always highly heterogeneous spatially and temporally. Open spaces such as streets, squares, plazas, grassland, and park for people's outdoor activities, generally exhibit differences in land cover (e.g., pavement, vegetation, water bodies) (51, 52), and surface structure (such as building height, street width, orientation, and density) (53, 54), resulting in the heterogeneity of microclimatic conditions and human thermal comfort. For instance, a square paved with cool materials could be 12°C cooler in surface temperature and 1.9°C cooler in peak air temperature (55). A study in Taipei indicated that parks could generate an average airborne cooling effect of 0.81°C in summer noon, and a cooling effect of 0.29 °C in summer night (56). Water bodies could be strong cooling sinks, compared with many other land use types (e.g. vegetation, road, building, bare land) (57). Overall, the heterogeneity of thermal environments generates significant implications for outdoor activities, transport, and heat health issues (58).

Existing studies have extensively reported the spatial heterogeneity of microclimates and outdoor thermal comfort. For instance, at the building scale, a study in Tianjin, China, indicated that daily maximum temperature decreased with building height at an average rate of 0.05°C for every 10 meters (59). However, the daily minimum temperature increased with building height at an average rate of 0.08°C for every 10 m. At the street scale, street orientation, aspect ratio, length-width ratio, sky view factor, enclosure degree, and building elevation can regulate aerodynamic and radiation properties and thereby the variation of thermal characteristics. A study conducted by Emmanuel et al. (60) in Colombo, Sri Lanka, found that air temperature reduced proportionally with the street aspect ratio and the distance from the sea, and the wind speed increased with the street aspect ratio (60). Street orientation affects solar radiation incidence. Ali Toudert and Mayer found that east-west streets were exposed to direct solar radiation much longer than other streets so thermal environments and thermal comfort in east-west streets were much worse (61). Taleghani et al. (62) analyzed summertime street thermal environments of a Netherland neighborhood, finding that east-west streets could receive solar radiation for 12.5 h, much longer than north-south streets (4.5 h). Moreover, the higher the sky view factor, the higher the mean radiant temperature (62). At the precinct scale, the thermal environments could be more complex due to the combined effects of building height, street structure, and building density. According to the local climate zone studies, the industrial and compact high-rise precincts could be much warmer than all other types. According to the precinct ventilation zone, open low-rise precincts in Sydney were the worst in PET, followed by open midrise and compact high-rise precincts (26).

Shading structures or tree shade could also result in strong cooling performance in the mean radiant temperature reduction (63, 64). Trees, in particular, could generate shades and evapotranspiration effects for better cooling performance. Existing studies indicate that though different tree species could have different cooling effects, the ambient temperature could still reduce by 0.5–1.0°C, and the reduction of mean radiant temperature was more prominent by 10°C (65). Local adoption of tree cooling strategies could increase the heterogeneity of street thermal environments since trees are not planted evenly in numerous precincts. In addition, waste heat or cold sources released from air-conditioning systems, traffic systems, and open doors and windows in and surrounding public spaces can also cause significant changes in local microclimate and thermal comfort (9). Overall, in heterogeneous neighborhoods, it is particularly important to predict and present microclimate and thermal comfort characteristics in a precise and real-time manner and to identify and determine areas with good microclimate and thermal comfort that are suitable for outdoor activities and daily travel for the analysis of FMC walkability.

### Transient and cumulative variation of thermal comfort

**Figure 2:**
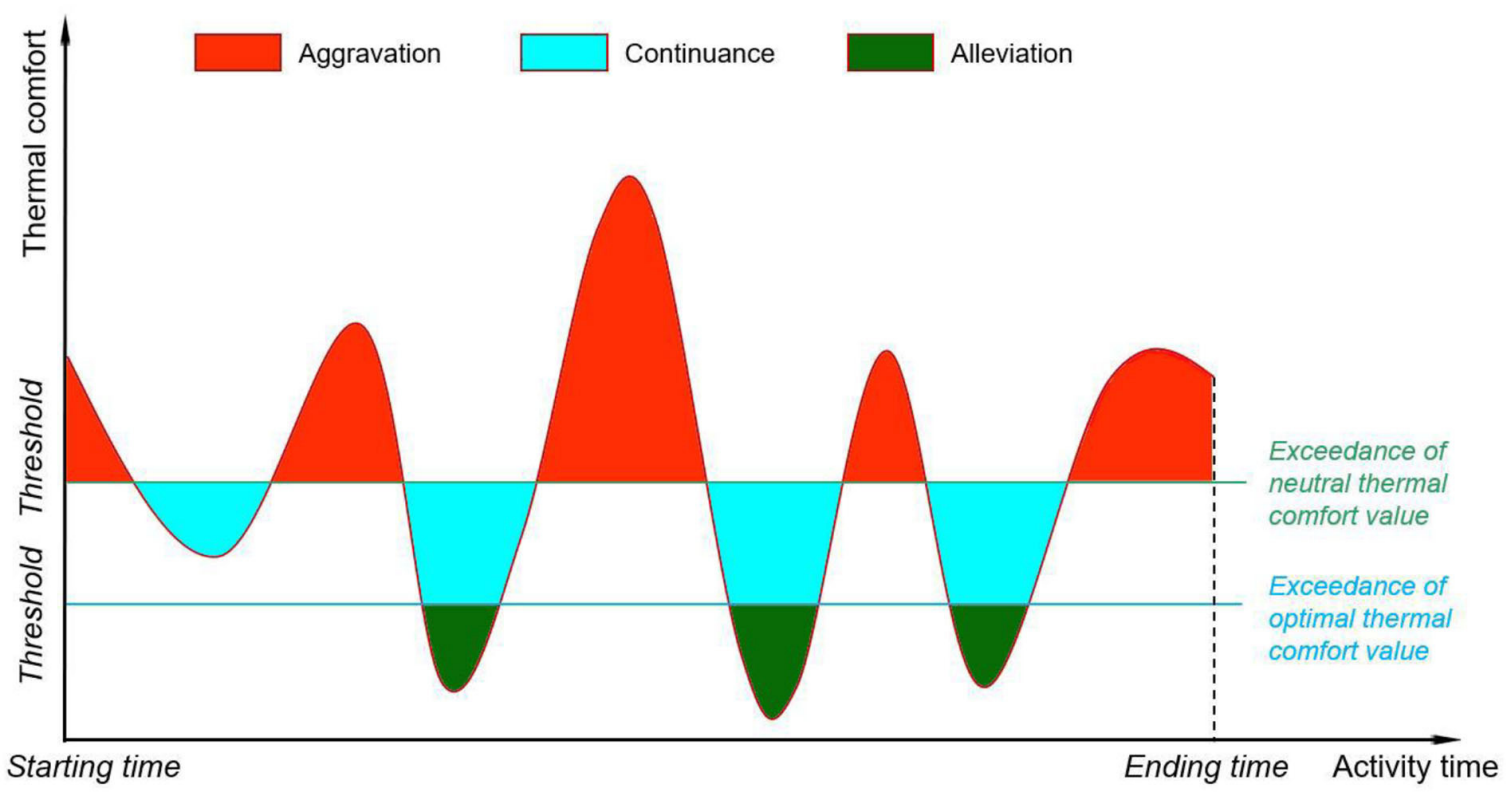
Heat stress aggravation, continuance, and alleviation with activity duration.

Since thermal comfort is the combined result of physical, physiological, and psychological responses, it exhibits spatial-temporal variations associated with a transient change of physical environment (40). Individuals can endure different levels of heat stress, due to the variation of mean radiant temperature, when walking in streets with sparse trees. During such a period, individuals may undergo a mix of conditions of heat stress aggravation, continuance, and alleviation (Figure 2), depending on the exceedance of the neutral heat stress threshold. If a thermal comfort value exceeds the neutral thermal comfort threshold, individuals will suffer from heat stress (Table 1), generating negative impacts on street walkability. Heat stress cumulates if individuals are walking in heat-positive environments continuously. In comparison, if a thermal comfort value is within or below the “no thermal stress” level, individuals will not endure heat-induced thermal stresses. The heat-stress-free level can be further divided into two sub-levels: (i) the heat stress alleviation zone, which alleviates cumulative heat stress, and (ii) the heat stress continuance zone, which neither alleviates nor strengthens the cumulative heat stress (Figure 2). Heat stress alleviation depends on the exceedance of optimal thermal comfort value.

Accordingly, individuals' bodies endure the alternation of stress aggravation, continuance, and alleviation when they conduct activities. Heat tolerance, namely the cumulative heat stress that reaches the maximum body adaptation capacity, is a function of aggravation, continuance, and alleviation (Equation 1).


(1) $$\begin{array}{l} \text{F}\left(x\right)=\text{f}\left(\text{aggravation}\right)\;+\;\text{f}\left(\text{continuance}\right)+\;\text{f}\left(\text{alleviation}\right) \end{array}$$

**Figure 3:**
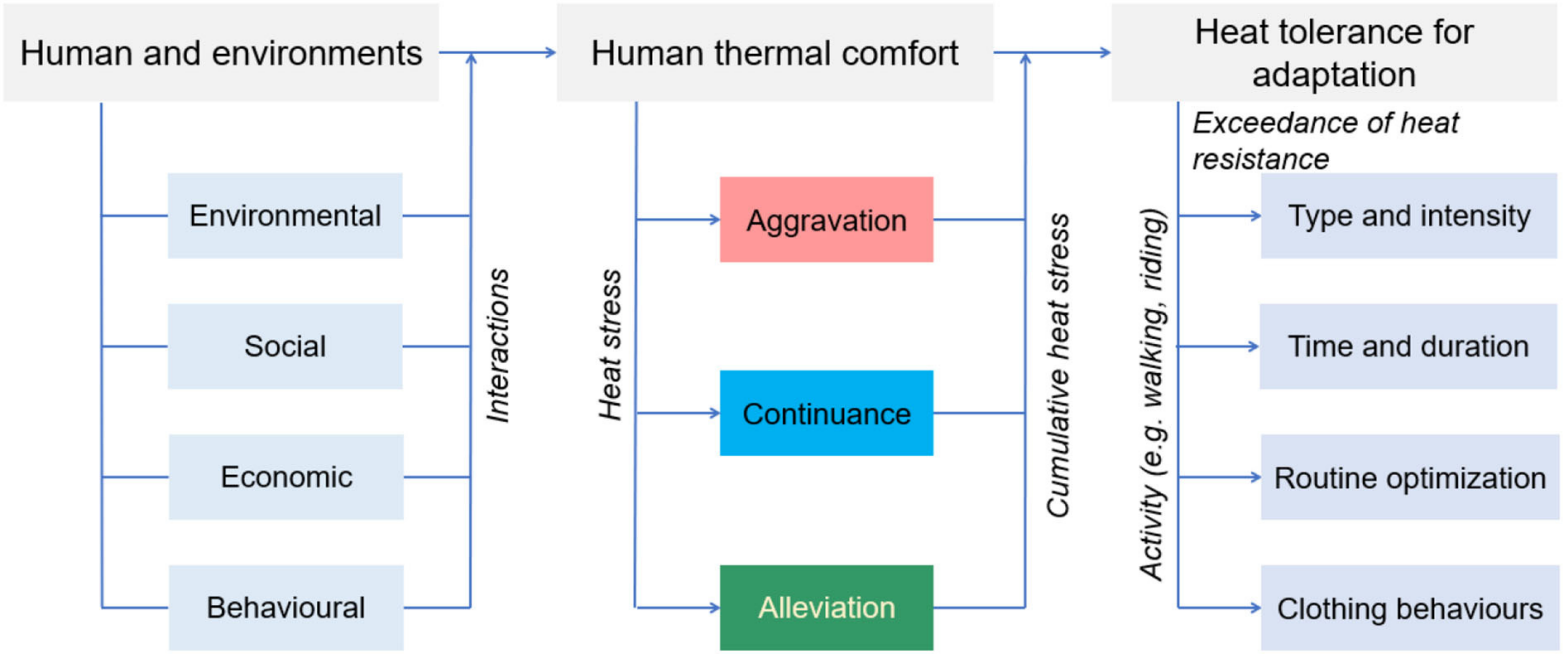
Association between heat tolerance and human-environmental characteristics.

Heat tolerance is also dependent on the socio-economic features of individuals (physiological response), their activity type and the alternation (behavioral feature), the clothing-related adaptive behaviors (behavioral and physical adaptation), their attention to thermal environments (psychological response), and the distance from the destination (social property). For instance, when individuals undergo severe heat stress and feel unwell during strenuous exercise, there is a high level of possibility of stopping exercising, meaning a change in heat stress aggravation to continuance or alleviation. Likewise, people may wear sun protection clothes or hold up an umbrella to shelter themselves from direct solar radiation, at which time the significant reduction of mean radiant temperature could indicate the transition from aggravation to continuance or alleviation. Limited time for commuting toward destinations (e.g., railway station, office, hospital), or visual and sound landscapes could divert their attention to thermal environments, resulting in increasing heat tolerance. Hiding in an attractive destination could potentially be an intervention of heat tolerance. Within an FMC, therefore, it is critical to consider associations between heat tolerance and social, economic, environmental, and behavioral characteristics for specific adaptation measures such as type and intensity, time and duration, route optimization, and clothing behaviors (Figure 3).

## Case study and method

### Case study area

**Figure 4:**
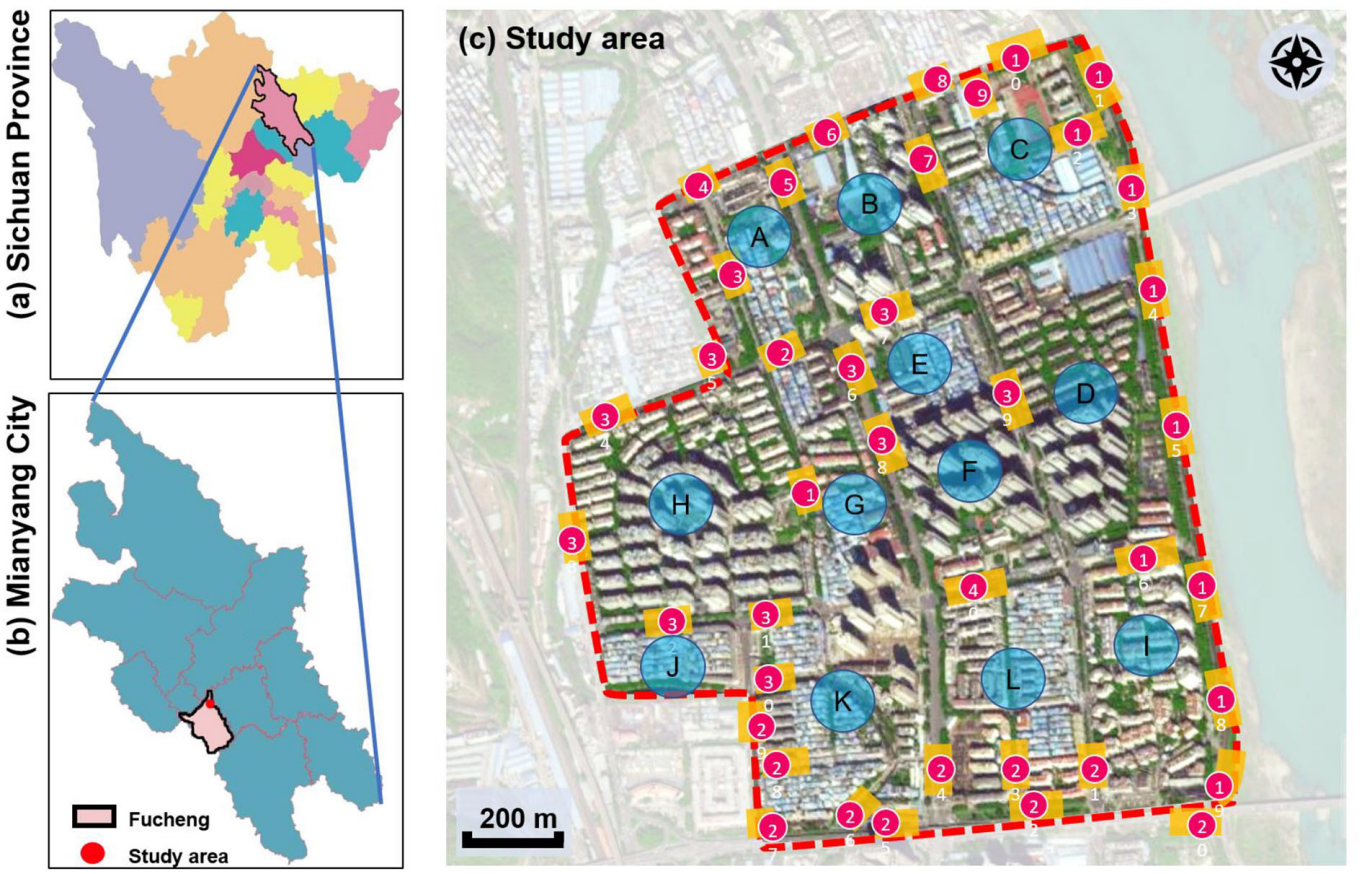
Location of study area and its streets and neighborhoods. **(a)** Sichuan province, **(b)** Mianyang city, and **(c)** Study area.

This study was conducted in Fucheng District, Mianyang, Sichuan, China. Mianyang (30°42′-33°03′N, 103°45′-105°43′E) is the second largest prefecture-level city of Sichuan province (Figure 4), with a total area of 20,267.46 km^2^ and a population of 4,868,243 according to the 2020 Population Census. Mianyang has an urban area of 2,755.4 km^2^ and an urban population size of 2,232,865. It has a monsoon-influenced humid subtropical climate (Köppen Cwa) with four distinct seasons. The annual average temperature ranges between 14.7 and 17.3°C. Its summer is hot and humid, whereas July and August are the two hottest months throughout the year, with average high temperatures of 30.1 and 30.5°C, respectively.

Mianyang city is undergoing rapid urbanization, where living quality and well-being have been important concerns given the urbanization experience and lessons from various highly urbanized cities. The 15-min city concept has been integrated into urban sprawl and urban renewal to meet residents' requirements for various public service facilities relevant to daily life, leisure, and entertainment. Furthermore, the FMC underlines the proximity of fundamental public services in the place. Such a principle requires not only the availability of public services but also the accessibility to them within FMC walkable areas. Therefore, the Gaoshui FMC in the Fucheng district was selected to analyze the walkability. This FMC consists of 12 neighborhoods (A–L, Figure 4), within which the buildings have various ages and streets exhibit different kinds of typology. On function type, the FMC is mainly used for residential purposes and is home to about 73,000 citizens. It also includes commercial, food, accommodation, and maintenance facilities for daily life. In particular, the flat terrain enables people to walk or ride in daily transportation functions, whereas the outdoor thermal environment is a precondition of walkability, especially in summer.

The spatial environments of the FMC exhibit a high level of heterogeneity, indicating the spatial variations of thermal environments (e.g. air temperature, wind speed, thermal comfort), and variations of walkability. Table 2 details the morphological information of 40 street scenarios presented in Figure 4c. For instance, Street 1 is well structured with mid-rise buildings, and it is almost covered by tree canopies and offers ample shade from trees and evapotranspiration cooling. Street 2 is equally well structured with dense high-rise buildings on one side and dense low-rise buildings on the other; however, there are no street trees and people can find relief only from narrow building shades. Street 11 is a riverside road with trees on both sides of the street, and the road is surrounded by a large patch of lawn. Street 24 is the main road of the city with high vehicular traffic, and trees on the street offer good and sheltered walkways protecting people from direct solar radiation. Street 26 is a pathway along with a city river and is covered by trees and an awning. The spatial variation of urban morphological characteristics is always coupled with temporal variations of thermal environments given the complex combination of time-based heat sources/sinks with morphological characteristics. Therefore, people can hardly have an intuitive assessment of street heat stress. This necessitates an accurate assessment of heat stress and associated walkability in this community.

**Table 2:** Morphological characteristics of 40 street sections.

**NO**	**Imagery information**	**Details**	**NO**	**Imagery information**	**Details**
1		Four driveway lanes, no non-motorized lane, wide walkways, street tree canopy (~0.20 m in truck diameter), dense midrise buildings on both sides	2		Six driveway lanes, two non-motorized lanes, narrow walkways, no street tree canopy, dense low-rise or midrise buildings on both sides
	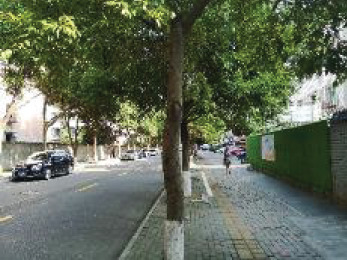			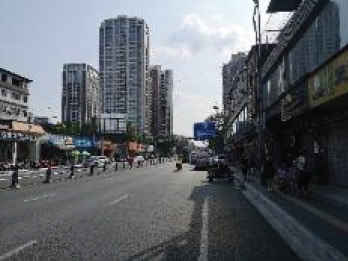	
3		Four driveway lanes, no non-motorized lane, wide walkways, street tree canopy (~0.30 m in truck diameter), dense midrise buildings on both sides	4		Four driveway lanes, no non-motorized lane, wide walkways, street tree canopy (~0.15 m in truck diameter), dense low-rise buildings on both sides
	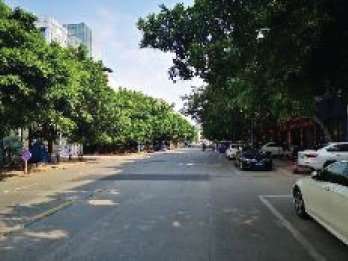			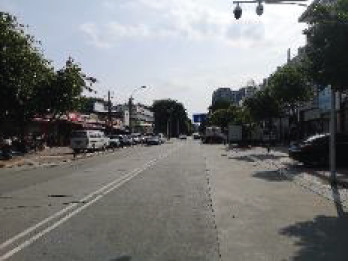	
5		Eight driveway lanes, two non-motorized lanes, wide walkways, street tree canopy (~0.25 m in truck diameter), dense midrise buildings on both sides	6		Four driveway lanes, no non-motorized lane, wide walkways, street tree canopy (~0.25 m in truck diameter), dense low-rise buildings on both sides
	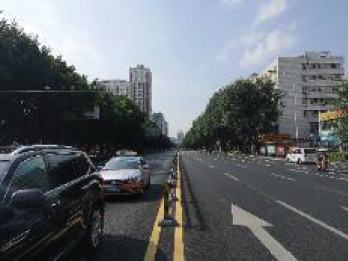			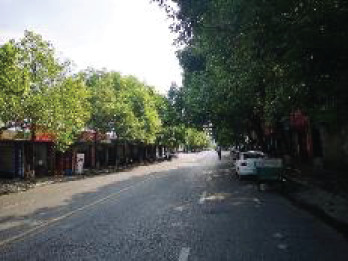	
7		Four driveway lanes, one non-motorized lane, wide walkways, street tree canopy (~0.15 m in truck diameter), dense low-rise buildings on one side and dense high-rise on the other side	8		Four driveway lanes, wide riding or walkways, street tree canopy (~0.15 m in truck diameter), dense low-rise buildings on one side and dense high-rise on the other side
	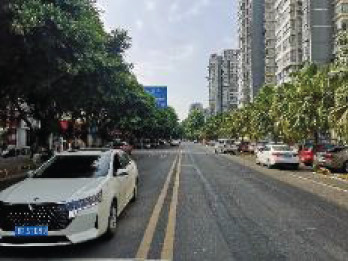			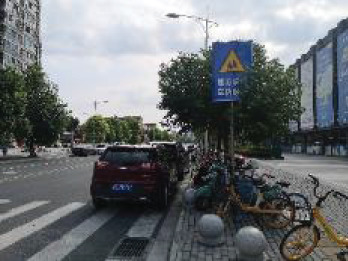	
9		Six driveway lanes, wide riding or walkways, sparse street tree canopy (~0.15 m in truck diameter), dense low-rise buildings on one side and dense high-rise buildings on the other side	10		Four driveway lanes, wide riding or walkways, sparse street tree canopy (~0.15 m in truck diameter), sparse low-rise buildings and lawns on both sides
	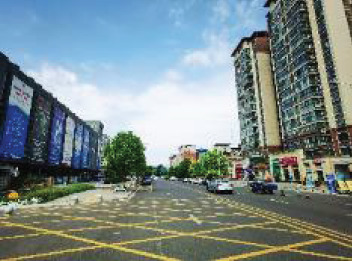			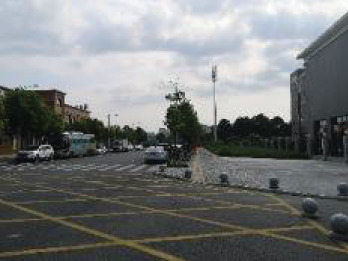	
11		Two driveway lanes, street tree canopy (~0.20 m in truck diameter), lawns and a narrow walkway on one side and a wide riverside walkway on the other side	12		Two driveway lanes, street tree canopy (~0.20 m in truck diameter), lawns and a wide walkway on one side, and a wide walkway and paved square on the other side
	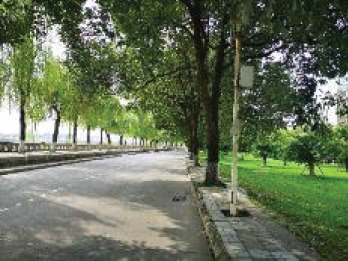			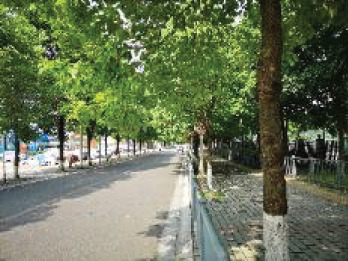	
13		Two driveway lanes, street tree canopy (~0.20 m in truck diameter) on one side, lawns and a narrow walkway on one side and a wide riverside walkway on the other side	14		Two driveway lanes, street tree canopy (~0.20 m in truck diameter), lawns and a narrow walkway on one side and a wide riverside walkway on the other side
	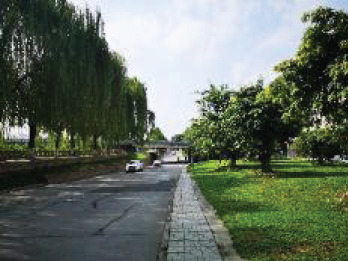			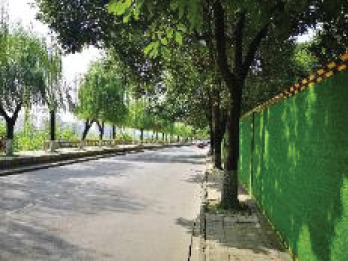	
15		Two driveway lanes, street tree canopy (~0.20 m in truck diameter), sunken lawns and a narrow walkway on one side and a wide riverside walkway on the other side	16		Four driveway lanes, wide walkways, street tree canopy (~0.20 m in truck diameter), dense midrise buildings on both sides
	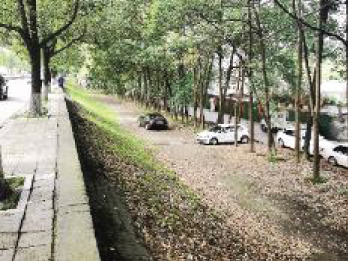			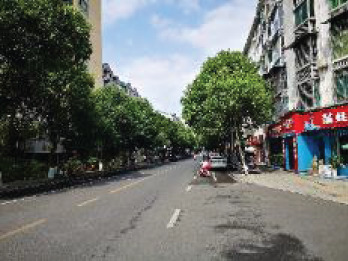	
17		Two driveway lanes, sparse street tree canopy (~0.20 m in truck diameter), sunken lawns and a narrow walkway on one side and a wide riverside walkway on the other side	18		Two driveway lanes, street tree canopy (~0.25 m in truck diameter), sunken lawns and a narrow walkway on one side and a wide riverside walkway on the other side
	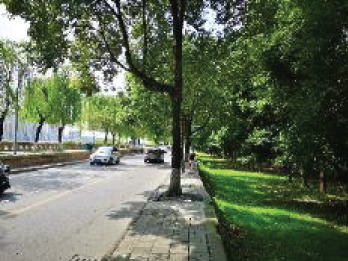			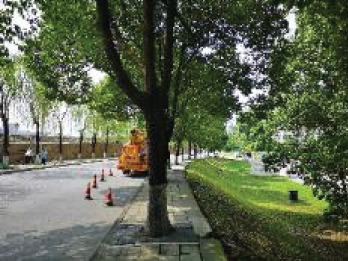	
19		Two driveway lanes, no street tree canopy, lawns and a narrow walkway on one side and an upper walkway on the other side	20		Two driveway lanes, street tree canopy (~0.20 m in truck diameter), narrow walkways on both sides
	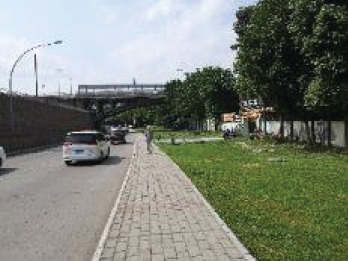			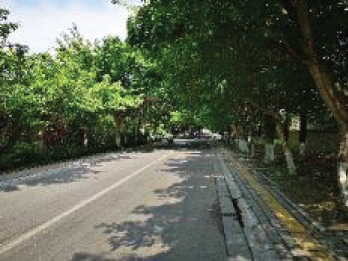	
21		Four driveway lanes, two non-motorized lanes, wide walkways, street tree canopy (~0.15-0.20 m in truck diameter), dense midrise buildings on both sides	22		Fastway under construction, wide walkways, street tree canopy (~0.25 m in truck diameter), dense midrise buildings on one side
	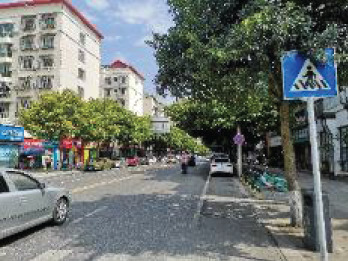			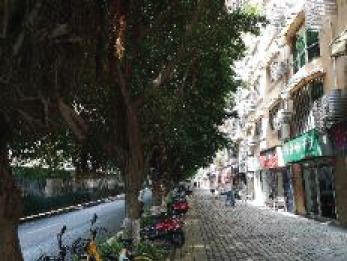	
23		Two driveway lanes, wide walkways, street tree canopy (~0.20 m in truck diameter), dense midrise buildings on both sides	24		Eight driveway lanes, wide walkways, street tree canopy (~0.15–0.20 m in truck diameter), dense low buildings on both sides
	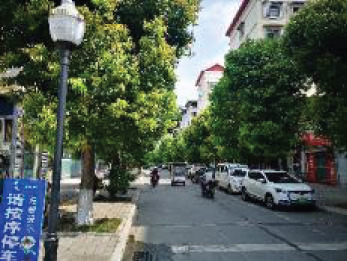			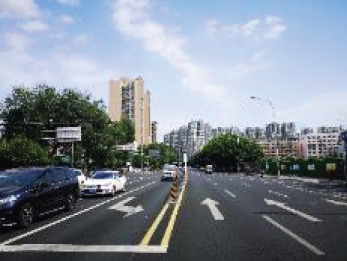	
25		Eight driveway lanes, wide walkways, street tree canopy (~0. 0.20 m in truck diameter), dense low buildings on both sides	26		A city waterway, wide walkways, street tree canopy (~0.25 m in truck diameter), building awnings.
	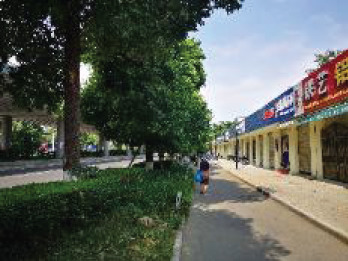			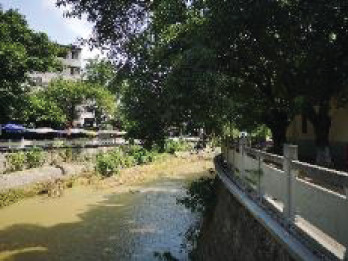	
27		Four driveway lanes, wide walkways, street tree canopy (~0.20 m in truck diameter), dense low- or midrise buildings on both sides	28		Two driveway/walkway lanes, no street tree canopy (~0. 0.20 m in truck diameter), dense midrise buildings on both sides
	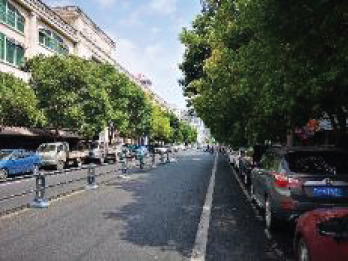			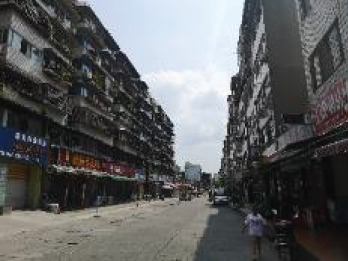	
29		Four driveway lanes, wide walkways, no street tree canopy, dense midrise buildings on both sides	30		A driveway, a wide walkway, street tree canopy (~0.25 m in truck diameter), dense midrise buildings on one side and a city waterway on the other side
	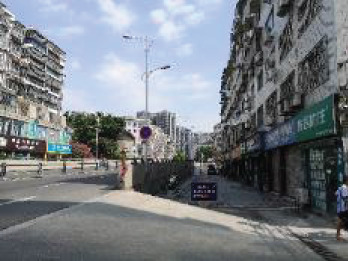			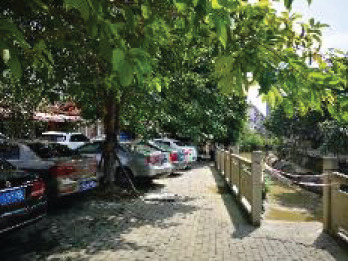	
31		Six driveway lanes, wide walkways, street tree canopy (~0.25 m in truck diameter) on one side, dense midrise buildings on both sides	32		Six driveway/ non-motorized lanes, wide walkways, street tree canopy (~0.20 m in truck diameter), dense midrise buildings on both sides
	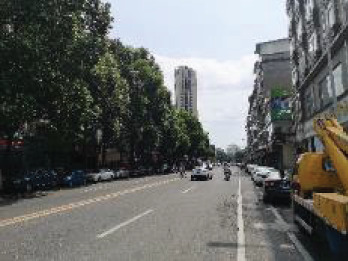			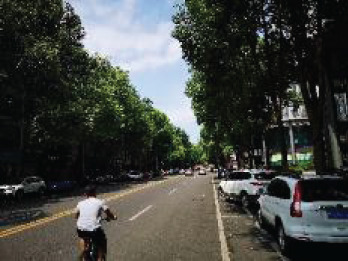	
33		Six driveway/ non-motorized lanes, wide walkways, street tree canopy (~0.15–0.20 m in truck diameter) on one side, dense low-rise buildings on both sides	34		Four driveway lanes, narrow walkways, no street tree canopy, dense midrise buildings on both sides
	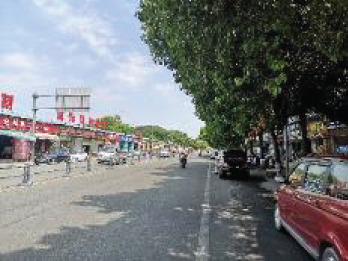			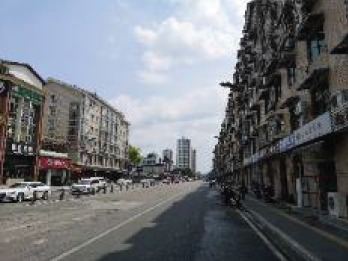	
35		Two driveway lanes, wide walkways, street tree canopy (~0.15 m in truck diameter), dense low-rise buildings on both sides	36		Six driveway lanes, wide walkways, street tree canopy (~0.15–0.20 m in truck diameter), dense midrise buildings on both sides
	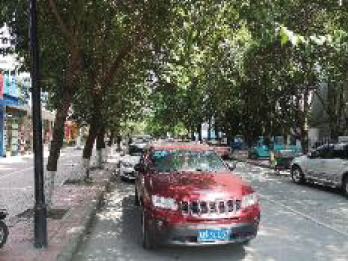			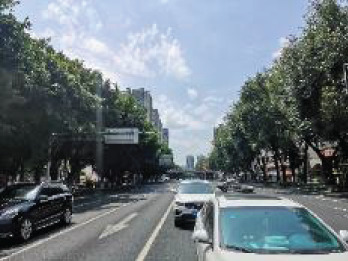	
37		Five driveway lanes, no street tree canopy, dense high-rise buildings on both sides	38		Eight driveway lanes, two non-motorized lanes, wide walkways, street tree canopy (~0.15–0.20 m in truck diameter), dense high-rise buildings on both sides
	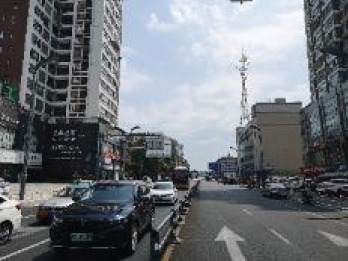			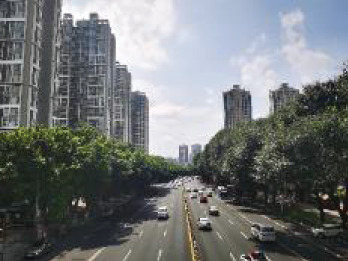	
39		Four driveway lanes, two non-motorized lanes, wide walkways, street tree canopy (~0.15 m in truck diameter), dense midrise buildings on both sides	40		Four driveway/non-motorized lanes, wide walkways, street tree canopy (~0.15 m in truck diameter), dense low-rise buildings on both sides
	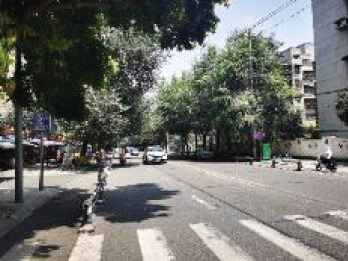			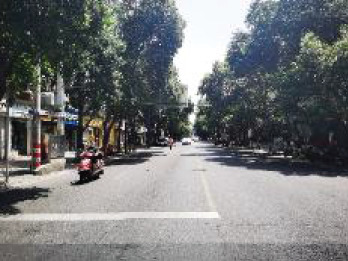	

### Thermal comfort assessment indicator and dynamic attenuation model

**Figure 5:**
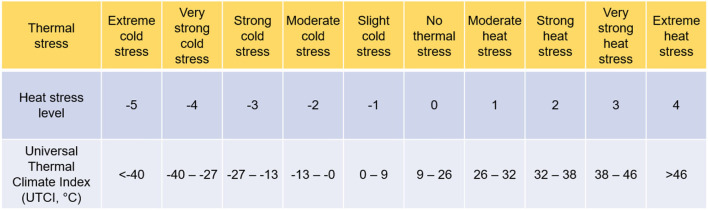
Association between heat stress levels and UTCI.

This study adopted the UTCI as the thermal comfort assessment indicator for it presents a multi-node model of human heat transfer and temperature regulation with the consideration of clothing adaptation. Furthermore, UTCI is more sensitive to the spatial variation of thermal environments, which is capable of simulating unsteady or transient conditions. Therefore, UTCI can well respond to the frequent spatial-temporal variations of the microclimates when walking and thereby reflect the dynamic psychological responses. The UTCI is also linked with different levels of thermal sensations (Figure 5), where a heat stress level (L_HS_) of 4 indicates an extreme heat stress level when UTCI is above 46°C.

To assess the dynamic variation of thermal comfort during walking, this study proposes a DAM of heat stress. The model defines the remaining tolerable heat discomfort (*R*_*t*_) during walking with the cumulation of heat stress. In a specific duration, the cumulation of thermal stress is defined as a function of time and heat stress level (Equation 2).


(2) $$S_{t}=\sum_{0}^{i}L_{HS_{i}}•t_{i}$$

where *S*_*t*_ is the cumulative heat stress with a time duration of *t* (Unit: $\overset{{\;}^{\prime }}{\min}$); _*L*_*HS*_*i*_ is the *ith* heat stress level, and *i* can be 0, 1, 2, 3, and 4; *t*_*i*_ is the time duration of the *ith* heat stress level (Unit: min).

Therefore, the remaining tolerant heat discomfort (*R*_*t*_) can be expressed as:


(3) $$\begin{array}{l} R_{t}=H-S_{t} \end{array}$$

where *H* is the maximum tolerant heat discomfort (Unit: $\overset{{\;}^{\prime }}{\min}$), at which value the remaining tolerant heat discomfort *R*_*t*_ equals to 0, indicating that people have to stop walking to relax. Moreover, it is suggested the higher the remaining tolerant heat discomfort, the higher the walkability of the street. In an FMC, citizens are expected to be informed of the route with the highest walkability.

To determine the maximum tolerant heat discomfort *H*, a 15-min walkability experiment was conducted in Fucheng District, Mianyang. During the experiment, the microclimatic parameters including air temperature, wind speed, relative humidity, and mean radiant temperature were recorded for analyzing the UTCI and weighting heat stress level. A total of 128 residents attended the experiment, where they were requested to walk for 15 min at four levels of heat stress (1, 2, 3, and 4). Their perceived heat stress levels (slightly uncomfortable, uncomfortable, very uncomfortable, extremely uncomfortable) were asked and recorded at an interval of 1 min. After the experiment, the reliability analysis was carried out using SPSS 22.0. The result shows a Cronbach's α coefficient of 0.929. The Cronbach's α coefficient of each variable was above 0.8, indicating the survey results were highly reliable.

**Figure 6:**
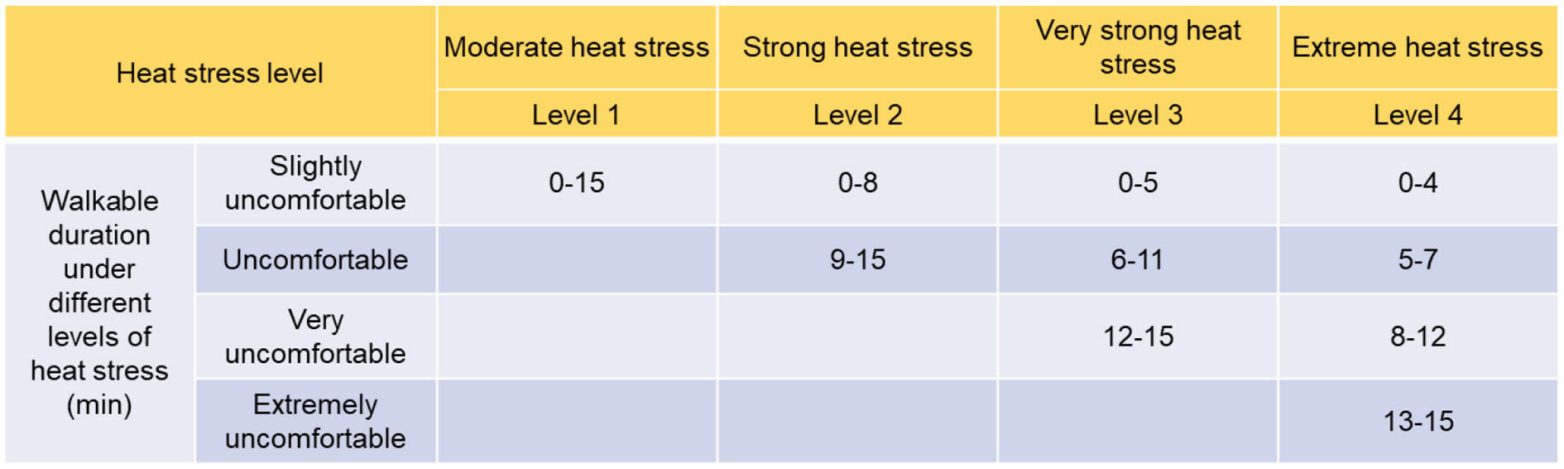
Heat stress level and its impact on walkable duration.

Figure 6 presents the walkable duration and respondents' discomfort level at different levels of heat stress. Respondents felt just slightly uncomfortable during their 15-min walking activity under moderate heat stress, and there were no uncomfortable, very uncomfortable, or extremely uncomfortable responses. In comparison, when respondents were under strong heat stress, they felt slightly uncomfortable in 0–8 min, while they expressed uncomfortable feelings in 9–15 min. Nevertheless, their feelings did not upscale to very uncomfortable and extremely uncomfortable levels. Under very strong heat stress, people felt slightly uncomfortable and uncomfortable in 0–5 min and 6–11 min, respectively. However, the cumulative heat stress made respondents feel very uncomfortable within 12–15 min. Under extreme heat stress, in comparison, people felt slightly uncomfortable in 0-4 min, uncomfortable in 5–7 min, very uncomfortable in 8–12 min, and extremely uncomfortable in 13–15 min. Therefore, a walkable duration of 13–15 min under Level 4 heat stress suggested a maximum tolerant heat discomfort *H* ranging between 52–60 min. In this study, an aspirational value of 60 min was considered. It should be noted that the walkable duration and associated maximum tolerant heat discomfort had already included physical, psychological, and physiological responses of respondents, rather than only considering physical environments.

Accordingly, the remaining tolerant heat discomfort (*R*_*t*_) is:


(4) $$\begin{array}{l} R_{t}=60-S_{t} \end{array}$$

### Numerical simulations of 15-min city thermal environments

To estimate spatial-temporal variations of thermal environments and associated heat stress, the ENVI-met model (Science version) was adopted. The ENVI-met model is currently the most holistic, three-dimensional microclimate model to present the high-resolution interactions among surfaces, plants, and the atmosphere. It has been widely adopted to calculate block- and neighborhood-scale microclimate and heat stress for climate-sensitive urban planning. The urban morphology, street network, tree planting and species, surface structure, and surface texture were obtained through satellite images, local urban planning council, and field observation. Given the large study area, we set 200 × 240 × 40 grids in horizontal, perpendicular, and vertical directions, with a grid resolution of 8 meters.

Weather condition is also important to simulate the ENVI-met model. The two hottest months of Mianyang are July and August. According to the data from China Meteorological Administration, an average temperature of 28°C was the most prominent in 2020 and mainly occurred in August. Therefore, typical weather conditions (wind direction, wind speed, air temperature, and relative humidity) were adopted for the simulation (Table 3). In the daytime, the temperatures were generally high and the walkable duration generally occurred between 8:00 and 19:00 h. In particular, the peak walking periods were 8:00–9:00 h and 17:00–18:00 h, when people go to and return from work. Compared with 8:00–9:00 h, the temperature at 17:00–18:00 h was much higher and heat stress was much worse, so the period returning from work was particularly analyzed.

**Table 3:** Weather data adopted to simulate the ENVI-met model.

**Data category**	**Detail**	**Data category**	**Detail**
Geographic location	30°42′-33°03′N, 103°45′-105°43′E	Minimum air temperature	24.6°C (07:00)
Simulation time	0:00–19:00	Maximum air temperature	32°C (15:00)
wind direction	245°	Minimum relative humidity	66% (17:00)
wind speed	1.4 m/s	Maximum relative humidity	94% (09:00)

Note that the above-mentioned simulation settings have been adopted to demonstrate the applicability of the DAM in walkable route planning. Comprehensive planning for a walkable route should be presented hour-by-hour, and the resolution should be higher to capture spatial heterogeneity of street sections, building heights, and tree heights. Moreover, to ensure people are better protected, an hour-to-hour local weather boundary condition, rather than the data from national weather stations which always fail to accurately present local climates, is needed to precisely capture the real-time maximum temperature and heat stress.

## Result analysis and discussion

### Verification of spatial heterogeneity of thermal comfort

**Figure 7:**
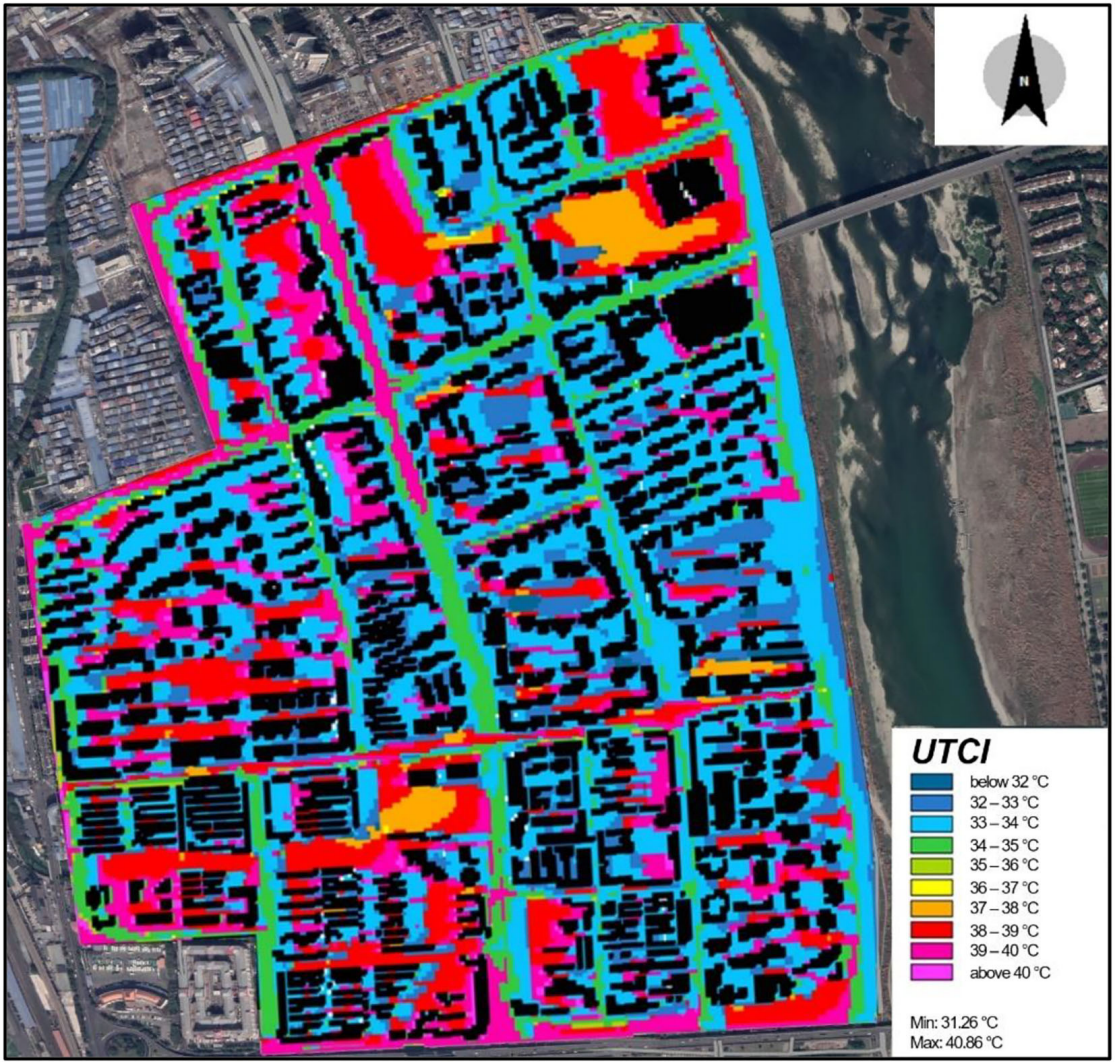
Distribution of simulated UTCI of the study area.

Figure 7 presents the simulated thermal comfort (UTCI) of the FMC between 17:00 and 18:00 h. The UTCI ranged between 31.26 and 40.86°C, primarily showing strong heat stress and very strong heat stress that could be categorized as Level-2 and Level-3 heat stress (Figure 5). The thermal comfort distribution was highly heterogeneous. Overall, the thermal environments within each neighborhood were much better than on the streets. This was mainly linked to building shades, tree canopies, and grassland within the community. Nevertheless, the neighborhoods were dense and enclosed, and in areas that were paved with cement and under direct solar exposure, the thermal environments were much worse. The riverside streets (Street 11–19) presented a good thermal environment with the UTCI ranging between 32 and 34°C. In comparison, north-south streets at 33–34°C exhibited a lower UTCI level than east-west streets at 36–40°C. The results are also dependent on the height of the buildings on the street. For instance, the middle section of streets 5 and 36 with low and mid-rise buildings had a higher UTCI level of 39–40°C than Street 38 with mid- and high-rise buildings at 33–35°C. Similarly, in streets 5 and 36, their central section was warmer than the sides due to the shade from trees and buildings. The results also indicate an open and wide street adjacent to the river could have a better thermal environment, for instance, Street 37 at 34–35°C.

### Assessment of street walkability

**Figure 8:**
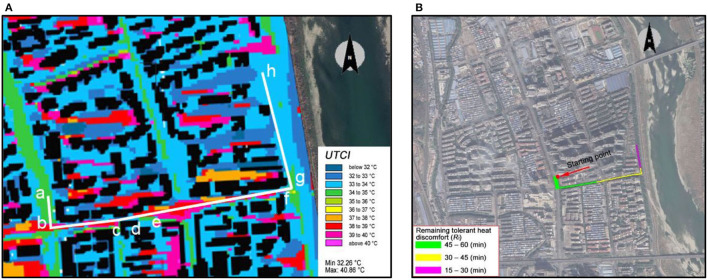
Street sections of a walking route in Neighborhood-F and the remaining tolerant heat discomfort. **(A)** Distribution of thermal comfort, **(B)** Walking route.

According to the municipal roads and land boundaries within the study area, 12 neighborhoods were identified (Figure 4). Setting the entrance of these neighborhoods as the starting points for daily walking, the walking routes were analyzed taking into consideration heat stress. The maximum walkable distance was set as 900 m based on a walking speed of 1 m/s. Figure 8A exhibits a possible walking route (a-h) of Neighborhood-F, (Figure 5), and the DAM (Equations 2,4), the remaining tolerant heat discomfort was calculated, as shown in Table 4.

**Table 4:** Dynamic variation of the remaining tolerant heat discomfort of a walking route of Neighborhood-F.

**Street**	**UTCI (°C)**	**L_HS_**	**Street distance (m)**	**Walking duration (min)**	**R_t_ (min^′^)**
ab	34–36	2	71.1	1.2	57.6
bc	38–40	3	171.7	2.9	48.9
cd	33–34	2	44.8	0.8	47.3
de	38–40	3	51.0	0.9	44.6
ef	37–40	3	292.0	4.9	29.9
fg	33–36	2	12.0	0.2	29.5
gh	32–36	2	257.3	4.3	20.9

The results indicate that section ab had a UTCI range of 34–36°C, namely a heat stress level of 2. Its street distance of 71.1 m, corresponding to a walking duration of 1.2 min, led to cumulative thermal stress of 2.4 min, and thereby a remaining tolerant heat discomfort of 57.6 min, based on the maximum tolerant heat discomfort of 60 min. Following this, from point-a to point-h, the remaining tolerant heat discomfort for 900 m was 20.9 min. As shown in Figure 8B, in particular, the section ae had an R_t_ of 45–60 min, section e. g, 30–45 min, and section gh had 15–30 min.

**Figure 9:**
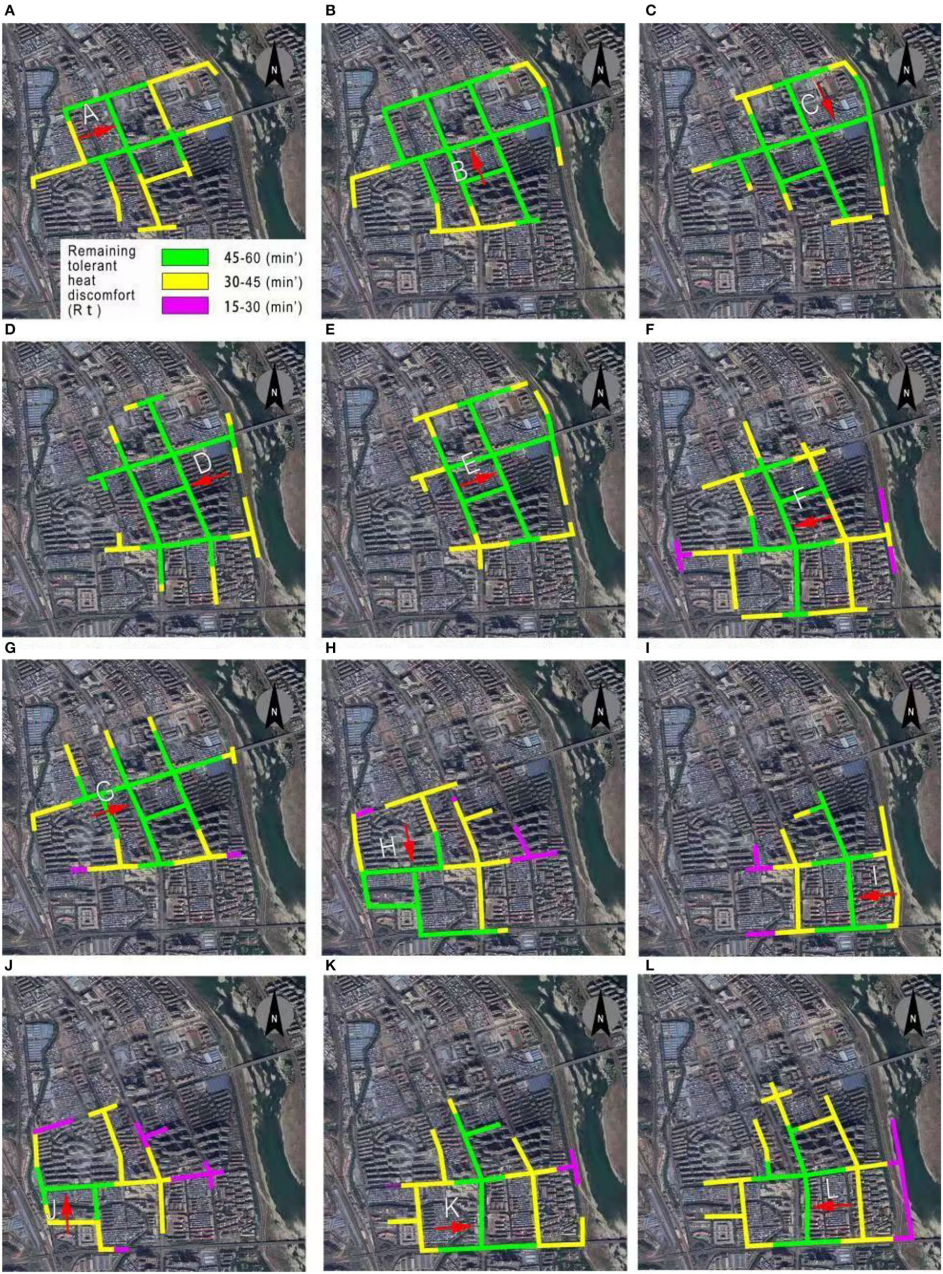
Identification of remaining tolerant heat discomfort of all possible walkable routes of 12 neighborhoods. **(A)** Neighborhood-A, **(B)** Neighborhood-B, **(C)** Neighborhood-C, **(D)** Neighborhood-D, **(E)** Neighborhood-E, **(F)** Neighborhood-F, **(G)** Neighborhood-G, **(H)** Neighborhood-H, **(I)** Neighborhood-I, **(J)** Neighborhood-J, **(K)** Neighborhood-K, **(L)** Neighborhood-L.

Likewise, 15-min walkable routes were identified, as shown in Figure 9. It was observed that the 15-min walkable routes of neighborhoods A, B, C, D, and E were mainly in green and yellow color, representing a remaining tolerant heat discomfort above 30 min. In comparison, other neighborhoods presented green, yellow, and purple colors, indicating a remaining tolerant heat discomfort above 15 min. Such a comparison further indicates that neighborhoods F-L were more vulnerable in the 15-min walkability. While such results could directly help identify the travel paths for citizens to avoid strong heat stress, the results also suggest that there is a need to adopt urban heat mitigation and adaptation techniques and strategies to improve heat resilience. Different from existing studies on dealing with heat stress by adding location-based interventions, the current walkability-based study provides new insights into planning-based adaptation. For instance, for the walkable routes in Neighborhood-F (Table 4), interventions added to improve thermal environments in a-h sections can potentially improve the path walkability by reducing the heat stress of any section.

### Assessment of the FMC walkability and implications

To support the FMC planning and design, this study further assessed the overall walkability of the study area. Based on street walkability (Figure 9), a population-based weighting algorithm (Equation 5) was adopted to adjust the heat stress of each street.


(5) $$k_{i}=\frac{P_{i}}{\sum_{1}^{n}P_{i}}$$

Where, *k*_*i*_ is the weight of each neighborhood in the 15-min city; *P*_*i*_ is the population number of each neighborhood; and *n* is the total number of neighborhoods, set as 12 in this study.

The weight *k*_*i*_ of each neighborhood is presented in Table 5.

**Table 5:** Weight of each neighborhood for adjusting street walkability of the study area.

**Block**	**A**	**B**	**C**	**D**	**E**	**F**	**G**	**H**	**I**	**J**	**K**	**L**
Population (k)	3.0	2.7	2.2	12.7	2.7	6.1	3.4	8.2	2.8	6.2	9.8	13.0
Weight *k*_*i*_	0.04	0.04	0.03	0.17	0.04	0.08	0.05	0.11	0.04	0.09	0.13	0.18

**Figure 10:**
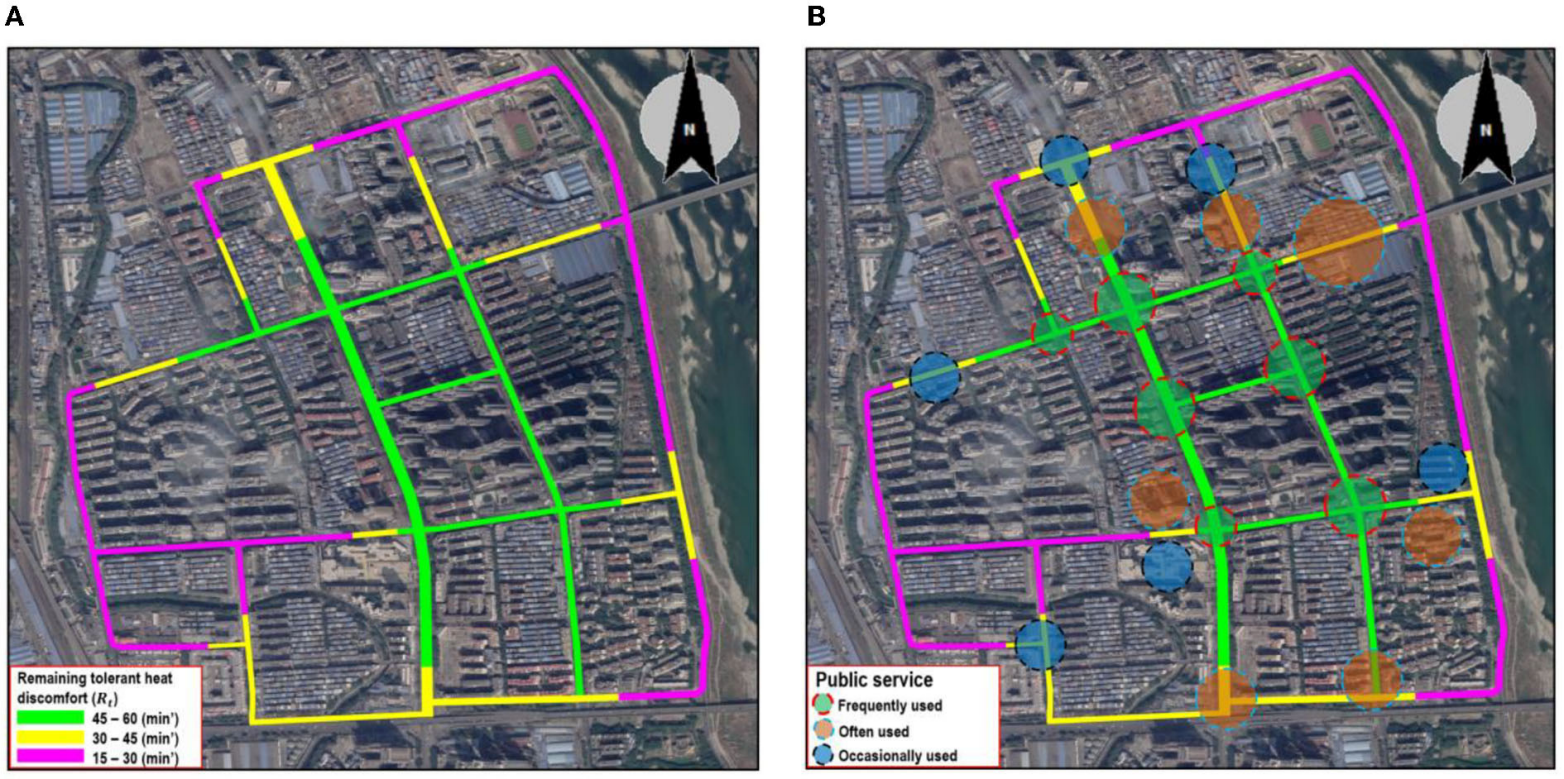
Overall street walkability of the FMC **(A)** and associated public service planning **(B)**.

The overall street walkability of the FMC, after taking into consideration heat stress, is generated in Figure 10A. Overall, the central part exhibited the highest remaining tolerant heat discomfort, while the outmost areas had the lowest remaining tolerant heat discomfort. To improve walkability, there is a need to focus on the improvement of thermal environments. In general, it is better to improve the walkability of the central parts (with the highest weight) to extend the overall walkability. However, in this study, the central areas generally had a low UTCI (Figure 7) and weak heat stress so adding interventions in the central parts did not exhibit high potential. Instead, adding cooling interventions in the yellow section could help weaken heat stress and extend street walkability the most. The improvement of the thermal environments of the purple section is also recommended.

The overall walkability also generates implications on the configuration of public service which is a typical adaptation measure to reduce citizens' heat stress by foot. The public service was divided into three types, according to the visiting frequency: frequently-used, often-used, occasionally-used, and indirectly-used (Table 5). In general, the indirectly-used public service is the one people do not visit directly or seldom visit, so it was not considered in this study. Figure 10B presents possible locations of frequently-used, often-used, and occasionally-used public services. The frequently-used public service was configured at the street sections with the highest remaining tolerant heat discomfort, and the occasionally-used public service was set at the street sections with the lowest remaining tolerant heat discomfort. The often-used one was set in the green-yellow transition street section or a green section. Our empirical work also found that various existing public services which are frequently used and often used were configured in yellow- and purple-colored street sections, making many citizens rely on private cars rather than walking. Therefore, urban heat mitigation strategies should be added to improve the thermal environment and street walkability. Simultaneously, public services should be gradually relocated to improve the accessibility of public services within walkable areas.

## Conclusion

The FMC concept has been implemented in various countries to build high-quality cities. Whilst the goals of inclusiveness, safety, and health have been widely accepted, the achievement of such goals is compromised since inclusiveness, safety, and health are interlinked with various factors. This study considered the issue of urban heat which is increasingly severe along with climate change and urbanization. This is a pioneering study on integrating heat-related impacts within the FMC framework by investigating walkability and developing a dynamic attenuation model (DAM) of heat stress. The model was applied to an FMC in Fucheng District, Mianyang, China. Some imperative results that were suitable for the case study have been obtained, and the results could generate implications for the (re)configuration of public services in urban planning and design. Overall, the findings could provide a practical reference for urban heat mitigation and adaptation. This paper has also meaningfully added new knowledge on heat-related FMC planning and design.

Whilst this study is the first one on the integration of heat-related walkability into the FMC concept, there are some limitations that should be considered. First, the remaining tolerant heat discomfort was developed based on the empirical study among 128 interviewees; the sample size might not be large enough to completely represent everyone's heat tolerance, and the empirical study had not integrated socioeconomic characteristics and public services that might intervene in people's heat responses for comprehensively assessing walkability. In particular, for vulnerable groups including children, older adults, and pregnant women, there should be an accurate heat stress threshold. Second, this study set four levels of heat stress for determining the remaining tolerant heat discomfort, while the constant heat stress could not present the variability of the thermal comfort and heat stress. As a result, the heat aggravation, continuance, and alleviation had not been well considered. Third, the model adopted the UTCI to assess the heat stress, while the capacity of other outdoor thermal comfort indicators such as PET, and WBGT had not been used in this study. Fourth, the study adopted an FMC in Fucheng as the case study. Whilst the results can provide strong implications for FMC planning and design, the applicability of the results for other cities might be uncertain since the clothing behaviors and heat-resistant capacity could vary with regions. Fifth, heat-induced impacts on thermal comfort happen throughout the day, especially in the daytime, while this study only considered the period between 17:00 and 18:00 h for walkability assessment and walkable route determination. Planning suggestions on public services were also given based on this time period, which could lead to biases if an urban design scheme was implemented based on the results presented in this paper. More efforts should be conducted to improve DAM accuracy, especially in social-economic, spatial, and temporal aspects, for broad applicability.

## Data availability statement

The raw data supporting the conclusions of this article will be made available by the authors, without undue reservation.

## Author contributions

YW: conceptualization, formal analysis, funding acquisition, investigation, methodology, software, validation, visualization, roles, and writing—original draft. B-JH: conceptualization, funding acquisition, project administration, methodology, resources, visualization, and writing—review and editing. CK and XC: investigation, methodology, and writing—review and editing. LY: investigation, methodology, and writing—review and editing. MY: investigation, and methodology. XL: conceptualization, formal analysis, and writing—review and editing. TZ: supervision, project administration, resources, and writing—review and editing. All authors contributed to the article and approved the submitted version.

## Funding

Project no. 2021CDJQY-004 was supported by the Fundamental Research Funds for the Central Universities; State Key Laboratory of Subtropical Building Science, South China University of Technology (Grant Nos. 2022ZA01 and 2021ZB16). Graduate Scientific Research and Innovation Foundation of Chongqing, China (CYB22058). Special thanks go to the National Natural Science Foundation of China (Grant No. 52108011); Guangzhou Philosophy and Social Science Planning 2022 Annual Project (Grant No. 2022GZQN14); China Postdoctoral Science Foundation (Grant No. 2021M701249); Department of Education of Guangdong Province (Grant No. 2021KTSCX004); Science and Technology Program of Guangzhou, China (Grant No. 202102020302); and Department of Housing and Urban-Rural Development of Guangdong Province (Grant No. 2021-K2-305243).

## Conflict of interest

XL was employed by Architectural Design and Research Institute Co., Ltd.

The remaining authors declare that the research was conducted in the absence of any commercial or financial relationships that could be construed as a potential conflict of interest.

## Publisher's note

All claims expressed in this article are solely those of the authors and do not necessarily represent those of their affiliated organizations, or those of the publisher, the editors and the reviewers. Any product that may be evaluated in this article, or claim that may be made by its manufacturer, is not guaranteed or endorsed by the publisher.
